# Aerosol Jet Printing in Biotechnologies

**DOI:** 10.3390/bioengineering13080918

**Published:** 2026-08-13

**Authors:** Pasquale D’Angelo, Giuseppe Tarabella

**Affiliations:** Institute of Materials for Electronics and Magnetism, Parco Area delle Scienze 37/A, 43124 Parma, Italy; giuseppe.tarabella@cnr.it

**Keywords:** aerosol jet printing, biotechnology, bioinks, tissue engineering, drug delivery

## Abstract

Aerosol jet printing, AJP, has emerged as a versatile direct ink writing technology enabling high-resolution, non-contact patterning of diverse biomaterials across a broad viscosity range. This capability facilitates the fabrication of complex micro- and mesoscale architectures on planar and non-planar substrates, advancing applications in biosensing, microfluidics, tissue engineering, and drug delivery fields. Herein, we review the integration of this high-resolution, rapid prototyping method with bioinks, including proteins, DNA, collagen, gelatin, and silk fibroin, and analyze how processing parameters influence structural and functional outcomes designed for applications for the above mentioned biotechnological fields. The ability by aerosol jet printing to combine structural, electrical, and biological functionalities within single platforms supports the development of multifunctional biomedical devices with higher potential than those produced using other direct ink writing techniques. While challenges such as bioink stability and process scalability, as well as the lack of deeper analyses about the efficiency of real applications of aerosol-jet-printed biotools, still remain open, AJP demonstrates significant promise as an enabling technology for next-generation biofabrication, offering new avenues for personalized and flexible biomedical applications.

## 1. Introduction

Additive manufacturing (AM) pertains to the set of fabrication protocols for the implementation of 3D-shaped artifacts by means of a layer-upon-layer approach. 3D printing methods, in particular, are widespread AM tools that, according to the scheme reported by Wong et al. [1], belong to the category of powder-based deposition processes in which the artifact manufacturing is promoted by binding mechanisms involving particle-based precursors. The development and evolution of 3D printing systems based on different types of precursors’ delivery mechanisms have led to a natural extension of the above scheme to liquid dispersions of particles (inks) or more viscous extrusion pastes.

Since the beginning of the 21st century, attention has been paid to 3D printing methods for fast prototyping protocols like the direct ink writing (DIW) approaches, which rely on non-contact techniques and exploit inks made of conducting precursors in the form of colloidal suspensions of particles, from a few to hundreds of nanometers in diameter [2,3], or are based on organic materials in solution [4,5].

DIW techniques are well suited for a ‘mask-less’ implementation of 3D-printed electronic devices [6], directly fabricated on a wide set of substrates, from conventional ones (Si/SiO_2_ wafers, conducting oxides indium tin oxide, ITO, and fluorine-doped tin oxide, FTO) to plastic supports (e.g., polyethylene terephthalate, PET, polyethylene naphthalene, PEN, polyimide, PI, and others). This is because DIW offers some peculiar advantages in terms of product design versatility, conjugating a marked flexibility for the allowed device architectures, a reduced materials consumption/cost, and a suitability for mass production of electronic devices and interconnects. Within this scenario, the most mature non-contact, DIW technique is inkjet printing (IJP). The IJP deposition process is based on electrostatic (piezoelectric and thermal) and electrodynamic actuators for the generation of ink droplets, at very high rate, that are directed towards the substrate once they are opportunely deflected at the nozzle outlet [7]. Although allowing rapid prototyping manufacturing, IJP suffers from a limited capability for the scaling down of printed features and an inability to deposit viscous inks, both reducing the set of deposable materials; for instance, IJP is suitable for the printing of inks with viscosities in the range from 20 to 40 cP [8]. A better alternative route, aerosol jet printing (AJP), overcomes such limitations, offering enhanced scaling-down capability and extending the viscosity range of printable inks [9]. Even though the final outcomes by AJP and IJP processes may be obtained by the same sequence of steps, i.e., the generation of ink flows made of droplets that are ejected from an orifice impacting on substrates placed on moving plates, such processes actually present subtle differences in terms of (i) the ink flow generation mode, (ii) the way in which the flow is delivered to the printing head and (iii) the ejection features at the nozzle orifice. Indeed, AJP allows the deposition of features with a higher line resolution, approaching in some cases a few microns [10], with a wider set of materials (viscosity ranging from 1 to 1000 cP [11]), and it is also suitable for depositing colloidal inks characterized by particles size ranging between 10 and 700 nm [2]. This printing method even envisages a reduced materials consumption as a consequence of the reduced volume-per-area delivery of ink relative to IJP [12]. It is worth noting that the higher viscosities of allowed inks permits enlargement of the AJP applicability range to 3D structures where in-plane and out-of-plane dimensions are comparable (3D structures, for instance, in a lattice form [13]).

Accordingly, AJP may act as an enabling process for several applications, inter alia: (i) flexible, bendable, conformable and wearable devices based on organic conductors and inks made of nanosystem suspensions [14,15,16]; (ii) integrated devices and (bio)sensors [17,18,19,20,21]; (iii) bio-inspired materials and devices [22,23,24]; (iv) energy conversion by photovoltaic paints (solar cells) and light-emitting devices repairing active layers in polymer solar cells [25,26,27]; (v) storage (batteries, supercapacitors) [28,29]; and (vi) integrated electronics (antennas and coils) for wireless powering/data communication [30,31,32]. Beyond 3D printed electronics, AJP allows very complex patterning and the definition of architectures on non-planar surfaces, while 3D IJP deals with low-viscosity materials and is therefore limited to producing artifacts with a narrow range of mechanical properties; this is particularly true for polymeric precursors in which low viscosity is related to shorter, less structured, polymer chains. In addition, among available DIW techniques, AJP is poised to become the one best suited to novel printing paradigms such as 4D [33], again thanks to the largest availability of printable materials.

Even if AJP shows enhanced 3D capability with respect to IJP [34], the technique is relatively young if compared to the latter; at present AJP is still not as popular as IJP, as also witnessed by the Web of Science database where the term “Aerosol Jet Printing” lists less than 1000 papers, nearly all published from 2006 to the present. Nevertheless, the literature already offers a wide-ranging description of the process features of AJP systems [9,35] and related issues (such as the overspray, OS, phenomenon) [15,36].

The potential of 3D printing by AJP has been explored also in applications for strategic sectors of biotechnology, such as tissue engineering, cell guidance and drug delivery (DD) [37,38]. The suitability of AJP for biotechnological applications stems from the high viscosity range of its printable inks, which enables the use of diverse material formulations and yields 3D structures with enhanced mechanical performance. This is particularly advantageous when printing on flexible or organic substrates or when engineering smart functionalities typical of 4D printing, such as electrical conductivity or shape-changing behavior, while still achieving high-definition 3D features [13].

Herein, some concrete examples and a critical evaluation of studies relating to the potential of AJP in bioprinting are reported and discussed in terms of advantages, limitations and perspectives. This analysis takes into account several aspects going beyond the mere description of processes and research products. It will consider aspects connected to materials as well, such as the features of inks realized so far.

While several reviews have addressed aerosol jet printing from a materials science or printed electronics perspective, focused analysis of its role in biotechnology, particularly in relation to bioinks, biological functionality, and translational applications, remains limited.

The primary objective of this review is to critically assess, through a narrative analysis of the available literature, the current state of aerosol-based jetting technologies in biotechnology. It focuses on applications in which AJP directly processes biological materials or contributes to biosensing, microfluidics, tissue engineering, cell guidance, and drug-delivery systems. Particular attention is given to the relationships among ink formulation, processing parameters, printed structure, and biological functionality, as well as to the advantages and current limitations of the technique. Bioelectronic devices interfacing with biological systems or living organisms are discussed where they provide relevant examples of structural, electrical, and biological integration, whereas purely electronic applications without a direct biological context are outside the main scope. The final aim is to distinguish established capabilities from proof-of-concept demonstrations and identify the requirements for broader biomedical translation.

The literature survey was conducted using the Web of Science, Scopus, and PubMed databases, covering publications from 2005 to 2026. Keywords included “aerosol jet printing”, “bioinks”, “bioprinting”, “biosensors”, “drug delivery”, and “tissue engineering”. Articles were selected based on relevance to AJP in biotechnology, with priority given to studies addressing biological validation and functional performance.

## 2. The Origins of AJP and Overview of Its Working Principles

The Mesoscopic Integrated Conformal Electronics (MICE) Program, promoted by the Defense Advanced Research Projects Agency (DARPA) in 2002, indicated the 3D printing technology as a new paradigm for mesoscale electronics. Thereafter, DIW techniques began to be exploited in the manufacturing of electronic components, such as passive and active components and devices [16,39], circuit manufacturing (i.e., logic gates, amplifiers or complex circuits) [40] and integration strategies allowed by the ease of printing devices/components and related interconnects. At first, IJP took advantage of the technological solutions developed for commercial desktop printers, as indicated by the above mentioned increment of literature documenting IJP use in research. Currently, although less explored than IJP, scientific production on the AJP technique has markedly increased since the first AJP system was launched on the market in 2004 by Optomec Inc. The review by Cooper et al. [41] traces the origins of AJP to the work by M. Renn, who holds the first patent of a prototype system for direct writing (2007). After joining Optomec (1999), Dr. Renn developed the modern versions of AJP systems under the DARPA indications; the first one was Maskless Mesoscale Materials Deposition (M3D), the subsequent evolutions of which are available under the trademark Aerosol Jet^®^. Nonetheless, concepts behind such techniques are considerably older. One of the first documented traces of studies dealing with the operation features of a 16-way home-made aerosol jet printing system dates back to the middle of the 1970s [42]. The documented activity is also supported by an analysis of the mechanisms at the basis of the fine-stream collimation and related enhancement of line resolution by AJP systems. The first applications on functional materials regard the manufacturing of ferroelectric lead zirconate titanate (PZT) films, reported in the middle of the 1990s by S. Kashu and coworkers [43,44]. It is from the second decade of the current century, nearly ten years after the precepts stated by the Mesoscopic Integrated Conformal Electronics (MICE) program, that AJP and electronics began to follow the same direction towards the establishment of an all-printed, large-area and flexible electronics. Pioneering studies, most of them disseminated at specific conferences on AM, show the suitability of AJP for 3D-printed electronics, implemented via deposition of electronic materials [45,46,47]. Bioinks have likewise been the subject of scientific studies dating back to the earliest investigations into the technique and its applicability [48], hence demonstrating that the use of AJP in the biotechnological sector was envisaged from the very beginning.

Currently, the introduction of novel commercial AJP systems, and the objective benefits it offers with respect to other DIW (including IJP) in terms of rapid prototyping of miniaturized devices or, the opposite, large area electronics, all make such a technique an emerging tool in the context of private and public research.

**Figure 1:**
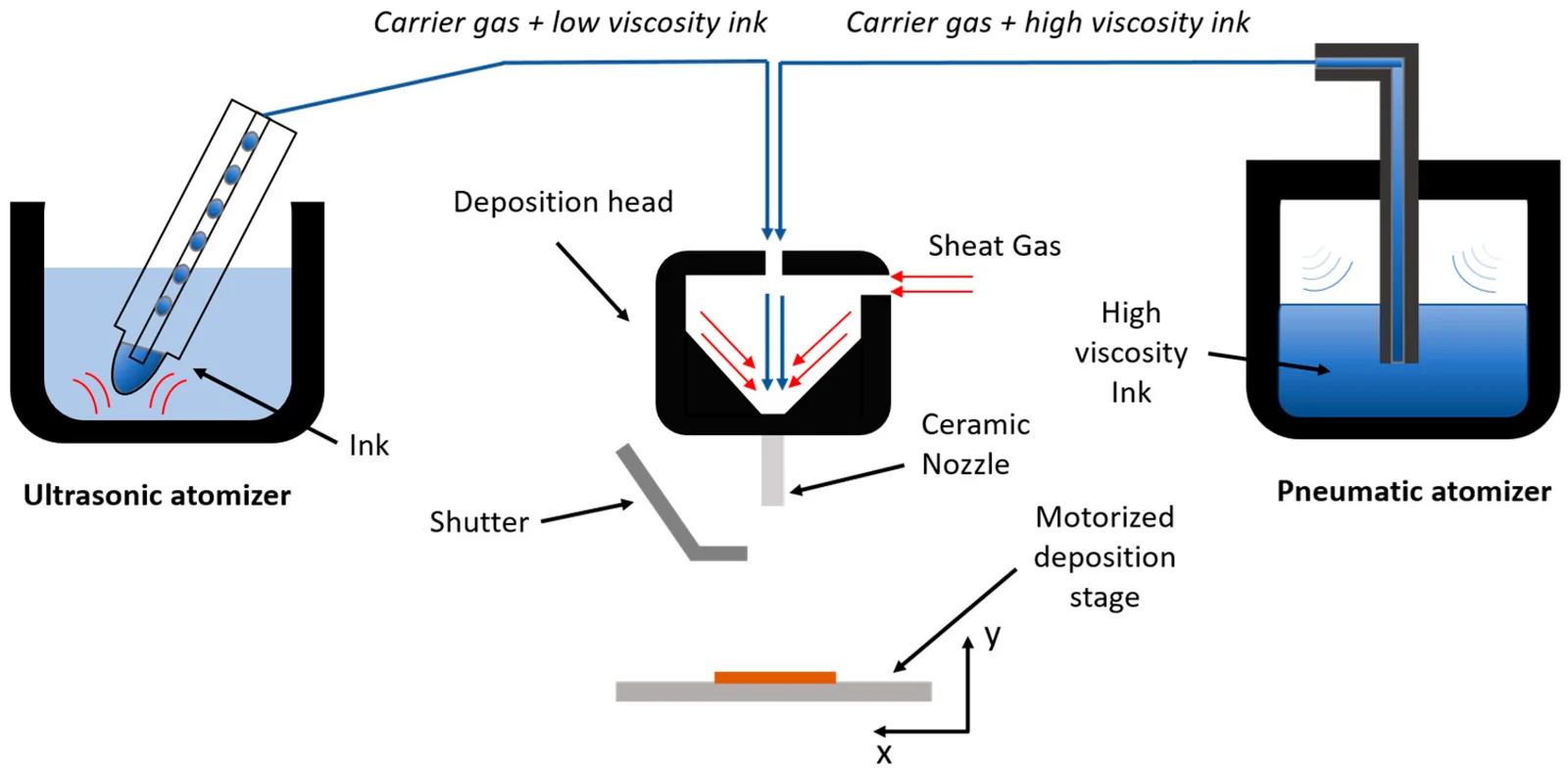
Schematics of the AJP process, from atomization to substrate delivery (adapted from [7], with permission of Springer Nature, Copyright 2022).

In the following, a brief description of AJP working principles is given, a schematic of which is reported in Figure 1. The ink delivery by AJP systems is ruled by the simultaneous action of two gas flows, i.e., the carrier and sheath gas flow (CGF and ShGF, respectively). The former acts on the transport of aerosolized inks in the pipeline connecting the atomizer generating an aerosol of micro-droplets, and the printing head (PH); the latter forces the generated aerosol along the central axis of the stream (collimation). At the basic level, AJP operation relies on two principles of atomization as the first step of the aerosolization process. AJP systems, indeed, generate an aerosol mist from ink droplets using either an ultrasonic (UA) or pneumatic (PA) atomizer. The aerosol is then transported and confined in a pipeline using the already mentioned CGF and ShGF, respectively, which help to enhance the velocity and focus the aerosol stream along the central axis to limit droplet spreading. UAs are suitable for low-density inks with viscosities between 1 and 10 cP, while PAs can handle inks with viscosities up to 1000 cP. This allows for high ink loading and conductivity in a single-pass deposition. Equipped with a nozzle implementing a controlled ink flow through its orifice, the PH focuses the ink stream within a nozzle, and the substrate is placed on a motorized deposition stage, a plate set at a fixed distance from the patterning source and controlled by software that converts computer-aided design (CAD) designs into vector-based tool paths. Worth noting is that the marked design versatility by DIW non-contact techniques, in particular AJP, essentially relies on the use of the CAD software tracing the patterns to be printed. As a process drawback, AJP line features suffer from the overspray (OS) phenomenon, a printing instability caused by droplet deposition outside the intended line profile, and suggested strategies to mitigate it, such as using larger nozzles and adding less volatile co-solvents to ink formulations, are offered by the literature [10,49].

Understanding these principles is essential to interpret the interaction between process parameters and bioink behavior, discussed in the next section.

## 3. Biocompatible and Bioinspired Inks

In a biotechnology context, AJP poses as a versatile, maskless, direct-write biofabrication technology that bridges biology and electronics, enabling localized biofunctionalization of sensor surfaces [50] and lab-on-chip (LoC) components [51], even on non-planar geometries. However, evergreen trends in biotechnology are DD and tissue engineering, and the connecting link between these research areas and the AJP technique is represented by bioinks allowing ultrasonic AJP for drug-testing platforms [52] and tissue-engineered constructs [53] in which controlled cell adhesion and organization are essential, even at the cost of somewhat lower edge resolution and coating uniformity.

The literature explores the use of 3D AJP for microstructuring biocompatible and bio-based inks, highlighting its potential in biotechnology through high-resolution deposition achieved by a fine tuning of printing parameters. Bioinks made of proteins, protein–metal hybrid composites, living cells and gelatins, as well as strategies combining AJP and other manufacturing routes to impart the desired function, are the subject of (albeit few) dedicated works in the literature since the early appearance of the AJP technique.

A pioneering study by De Silva et al. introduced a two-step precision spraying method to micropattern living cells on both planar and complex curved substrates using focused jets of aerosolized polymer solutions [48]. In their approach, a polymer solution (adhesive, such as laminin or polyethylenimine (PEI), or non-adhesive, such as polytetrafluoroethylene, PTFE, or polydimethylsiloxane, PDMS) was aerosolized, focused with sheath gas through a 150 μm orifice, and written under CAD control to form about 25 μm-wide patterns. Cells were then simply plated, and selectively adhered only to permissive regions. Positive patterning was achieved by spraying adhesive biopolymers onto non-adhesive PDMS so that diverse cell types formed stable patterns in serum-containing media, while negative patterning was performed by spraying hydrophobic polymers onto glass so that cells occupy only the exposed glass stripes, including on tapered capillaries and other curved supports. This study is a clear demonstration of how spatially selective surface properties permitted by the direct application of coatings with AJP represent in fact one of the major advantages of this technique when related to biotechnologies, in particular for scaffold coatings in tissue engineering.

A very recent work by Zhou et al. reports on a single-step aerosol jet co-printing method to fabricate flexible, conductive collagen/silver electrodes, unveiling the potential of co-printing to get hybrid biopolymer–inorganic materials whose characteristics are adjustable directly in line, not by preparing novel ink solutions [54]. The authors aimed at creating biocompatible, mechanically compliant, and electrically functional bioelectronic interfaces suitable for implantable devices. They demonstrated that by co-depositing collagen and silver nanoparticle (Ag-NP) inks and optimizing curing conditions, they could produce electrodes with low resistivity (~10^−6^ Ω·m), maintain conductivity under mechanical flexure, and partially preserve collagen structure for weeks in physiological media. Indeed, adjusting material ratios and processing parameters allowed tuning of electrical and biological performance, revealing a processing window where moderate cytotoxicity could be mitigated by higher collagen content, although long-term biocompatibility still remains a concern.

Focusing specifically on the use of true bioinks, Grunwald et al. showed that AJP can deposit microscale protein, DNA and enzyme patterns, as well as conductive metal features, on planar and complex 3D substrates without loss of biomolecular function [55]. They formulated bioinks from fluorescently labelled bovine serum albumin (BSA), DNA ladders and horseradish peroxidase, generated aerosols via ultrasonic or pneumatic nebulizers, and focused droplets with a coaxial sheath gas to produce 10–20 μm lines and spots at densities of ~1600 spots/mm^2^, while controlling overspray by adjusting ink viscosity (e.g., by adding ethylene glycol) and gas flows. They further demonstrated that high-molecular-weight DNA is damaged by ultrasonic atomization but preserved with pneumatic nebulization, that horseradish peroxidase (HRP) remains active after printing and mild heating (up to ~80 °C), and that silver nanoparticle inks can be printed and sintered into conductive interdigital electrodes (~25% of bulk Ag conductivity), into which micrometric BSA patterns are subsequently integrated using the same system.

Williams et al. investigated the use of ultrasonic AJP to deposit biological inks, specifically proteins like Cy5-labeled bovine serum albumin (BSA), streptavidin and anti-carcinoembryogenic antigen (anti-CEA) antibody, onto nonfouling poly(oligo(ethylene glycol) methyl ether methacrylate) (POEGMA) polymer surfaces (printing parameters: 100 µm nozzle, ShGF and CGF of 16 sccm, printing plate speed and temperature 0.5 mm/s and 30 °C, respectively) for in vitro diagnostic (IVD) applications [56]. Assay performance was used to demonstrate that ultrasonication does not degrade DNA and proteins or impair their biofunctionality. They found that AJP can produce sensitive immunoassays with sub-nanogram per milliliter detection limits and can print complex, multimaterial structures, including biological reagents and conductive nanowires, in a single process. These findings expand the potential of AJP for fabricating low-cost, integrated biosensors, revealing that ultrasonication does not necessarily damage proteins, as previously thought. Beyond serving as a process to pattern both biological recognition elements and electronic/microfluidic structures, which is powerful for next-generation biosensors, lab-on-chip systems, and bioelectronic devices, protein-compatible AJP represents a route towards the implementation of tissue models for lab studies (e.g., organ-on-chip) and applications in tissue engineering.

The studies herein presented demonstrate that by balancing UA and PA parameters, proteins of varying complexity, including antibodies and DNA, can be effectively deposited, finding applications in DD (e.g., antibody-mediated targeting [57,58]), tissue engineering, and biosensing (e.g., DNA sensors [59,60]).

While recombinant proteins and DNA-based biotechnologies offer precise control in biomedical applications, gelatin (derived through thermal hydrolysis of animal collagen) provides a natural, biocompatible biomaterial with similar structural roles in controlled release via hydrogels in DD and in 3D scaffolds for tissue engineering, as well as in medical devices like hemostatic sponges, and gelatin methacryloyl (GelMA)-based bioinks for 3D printing and biosensors [61]. The analysis presented by Phuah et al. similarly underscored how various printing parameters influence the resolution and quality of biomaterial lines produced by AJP, using gelatin as a model ink [62]. The authors systematically varied seven key parameters such as CGF, focusing ratio (FR), print speed, stage temperature, nozzle diameter, working distance, and ink concentration to understand their effects on line width, height, and features like drop formation and the coffee ring effect. They found that these parameters primarily affect processes like material deposition, evaporation, and aerosol focusing, with optimal ranges enabling the printing of gelatin lines as narrow as 30 μm. Interestingly, the systematic change of the above parameters produces expected results for CGF (i.e., increased line width and height, but also more overspray at higher GCFs), FR (i.e., too high FR increased overspray and loss of edge definition), platen temperature (i.e., higher temperatures resulted in more uniform line cross-section, but excessively high temperature promoted overspray or other defects), nozzle diameter (i.e., smallest nozzle realized the best combination of line height and width) and working distance (i.e., increased working distance corresponded to reduced resolution due to focusing loss). On the contrary, deviations from outcomes typically found in the printing of polymers and metallic inks were found in printing speed and ink concentration; in fact, an increased print speed only induced line height reduction (less material per unit length) upon keeping line width almost unchanged, while variations in concentration produced no significant dependence of line quality.

It is noteworthy that, after deposition, gelatins exhibit pronounced swelling, so even though line width typically decreases with higher deposition speed in AJP, the observed invariance thereof with respect to printing speed may arise from a competitive interplay among process parameters such as the deposition rate, the subdued temperature of the printing stage, and the therein used substrate’s wetting properties. However, beyond the mechanisms (which clearly warrant deeper investigation), the invariance of line width in favor of an increased line thickness as a function of printing process speed represents a significant advantage, at least in the case of gelatins, for implementing processes capable of yielding constructs with marked size in the out-of-plane direction.

Silk fibroin (SF) is another natural protein-based biopolymer widely used in biotechnology, in particular in applications for tissue engineering [63,64] and DD [65,66]. SF exhibits distinctive (bio)chemical and physical properties, featuring a high density of amino-acid functional groups and remarkable, easily tunable mechanical behavior achieved through simple and rapid strategies [67]. Despite the large literature on SF deposition by AM routes such as extrusion-based 3D bioprinting [68,69], 3D jet printing [70], binder jetting [71] and wet spinning/electrospinning [72,73], little has been done in the context of DIW; the literature offers just some insights related to IJP [74,75]. This is mainly due to the inherently low printability of bare SF when used as ink.

In the context of AJP, Xiao et al. developed a modified material formulation process, emphasizing precise control of degumming, dissolution, and dialysis steps, to produce stable regenerated SF solutions suitable for AJP [76]. They identified a narrow processing window defined by ShGF and CGF rates minimizing defects like overspray. Therein, the ShGF rate was varied between 20 and 150 sccm, with a maximum CGF of 50 sccm (platen temperature and speed 25 °C and 2 mm/s, respectively), finding an optimum focus ratio (i.e., ShGF/CGF) between 3 and 4 in terms of lower overspray width in all cases and a lower line width of about 10 µm for a CGF rate of 23 sccm. The as-shown findings demonstrated that optimizing these parameters allows overcoming the challenge of printing SF solutions without the use of additives like reagents or other biocompounds. This enables the fabrication of microscale SF patterns with improved resolution while providing new insights into the processability of biopolymer solutions via AJP, even without using additives.

Considerable challenges arise in achieving macroscopic SF structures by AJP, which are however theoretically feasible with wide-head AJP systems; however, the low thickness attainable with a single deposition pass necessitates a prolonged deposition process, which is dictated by the number of layers within the layer-upon-layer approach required to extend the size of the bulk system to the third dimension. Once again, designing appropriate formulations seems to be the route to follow in order to achieve SF materials with remarkable bio/chemical/physical properties and high printing quality using AJP. In short, through different formulations of a given printable ink and/or opportune deposition parameters, it is possible to tune the dimensional characteristics of the printed features [77].

### Collagen-Based Bioinks

A recent systematic review by Stepanovska et al. shows that collagen is widely used as a base hydrogel in cell-containing bioinks, often employed in extrusion, or laser-based printing systems, especially when high-fidelity scaffolds with good cell viability are sought [78]. Collagens are indeed the most extensively examined biomaterials in 3D bioprinting, owing to their biocompatibility, ability to mimic the native extracellular matrix (ECM), and seamless integration with cells and other biomaterials. For this reason, collagen bioinks deserve dedicated coverage in an independent section.

Since its emergence in the research field, AJP has proven useful as a method to produce dense, mechanically robust collagen scaffolds suitable for tissue engineering applications, particularly in replicating dense collagenous tissues like the cornea and cartilage. In this respect, Gibney et al. investigated AJP of collagen type I and II to fabricate dense, multilayered constructs with mechanical properties comparable to native collagenous tissues, particularly cartilage [79]. The authors showed that layer-upon-layer AJP can produce up to hundreds of stacked layers with effective elastic moduli in the 0.1–2 MPa range, placing printed collagen within reach of human ear, septal and alar cartilage stiffness, and significantly above corneal stroma. They highlighted collagen type II as especially suitable for AJP because its lower viscosity enables a wider printable concentration window and it is less susceptible to process-induced denaturation, while type I shows gradual structural damage under prolonged printing. Scanning electron microscopy (SEM) analysis reveals well-defined, continuous layers using an optimized printing process reducing the partial re-solubilization and the arising of amorphous regions and “pothole” topographies found in preliminary tests. Therefore, AJP emerges as a promising high-resolution biofabrication method for collagen-based tissue engineering and potential integration with conductive inks. However, drawbacks in scalability, process-induced denaturation and the need for careful control of printing/neutralization conditions similarly emerged from that study.

In a follow-up study involving some of the authors involved in the above discussed study, they also demonstrated the AJP printing of recombinant human collagen type III (RHCIII) with high resolution and forming multilayered, dense films [80]. Using a ShGF of 60 cc/min, and a CGF of 40 cc/min (deposition speed 11.5 mm/s), the authors fabricated AJP RHCIII constructs exhibited optical transparency with an average transmission of 87.5% across all visible wavelengths and refractive index of 1.35, which are comparable to native cornea, while mechanical characterization showed an average effective elastic modulus of 506 ± 173 kPa, significantly higher than previously reported values for pure collagen. As-found features fall within the range of human corneal tissue, despite some collagen denaturation having occurred during printing. These findings suggest that AJP can produce more mechanically robust and optically suitable collagen scaffolds than previous methods, providing insights into the potential of recombinant collagen bioprinting for corneal applications.

On the other hand, the work by Nair et al. focused on the use of AJP to enhance the piezoelectric properties and control the surface potential of collagen inks. The study explored the use of both pneumatic and ultrasonic AJP atomization methods to print Type I and Type II microfibrillar collagen from different anatomical sources [81]. Key findings of that study are: (i) the characterization of the rheological properties of the collagen inks showing that they behave as fluids at high frequencies, which is relevant for the ultrasonic AJP; (ii) the high-quality collagen lines (influenced by the CGF and ShGF rates) with minimal overspray and homogeneous coverage in an optimized deposition process; (iii) better alignment and lower surface roughness for ultrasonic AJP, which also did not cause notable denaturation; and (iv) the piezoelectric response of AJP-printed collagen significantly higher than that of drop-cast films, with the Type I collagen outperforming the Type II one. Although differences are found for the two aerosolization methods, the potential of AJP in terms of allowed materials and superior properties of as-deposited features are therein evident.

AJP deposition to fabricate collagen-based micro-structures for bone tissue engineering also has been explored; Degryse et al. characterized formulations of collagen type I inks incorporating hydroxyapatite (HAp) to mimic bone composition, and they assessed the impact of ultrasonic atomization on collagen integrity [53]. They found that while certain collagen-HAp inks could be successfully printed into 3D hollow pillars (19 ccm CGF, 40 ccm ShGF, printing speed of 0.4 mm/s, platen temperature of 30 °C to achieve high-resolution printing of ~20–50 µm, through inflight jet), the ultrasonic atomization caused collagen degradation. Strategies to overcome this drawback like the addition of cellulose nanocrystals (CNCs) actually have induced cytotoxicity, as shown by in vitro tests on MC3T3 cells.

Although studies consistently affirm the feasibility of collagen-based AJP, critical drawbacks including biocompatibility concerns, unpredictable degradation profiles, and laborious cross-linking protocols still persist. This underscores the need for meticulous optimization, particularly via refined ink formulations (whether pure collagen or related composites) to deliver stable systems that are conducive to effective 3D printing, but also via a proper tuning of process parameters.

Table 1 summarizes the composition, printing features and biological outcomes of the above described bioinks, while also reporting related limitations. The interplay between material properties and processing conditions directly impacts biological performance and application outcomes, as discussed in Section 4 and Section 5.

## 4. From Micrsostructuring Approaches to Applications in Biotechnology

Evidence from the literature highlights the feasibility of AJP-enabled microstructuring for biotechnological application. Microstructuring enables precise control over scaffold architecture at micrometric scales, enhancing cell adhesion, guided proliferation, dictating cell behavior through topography and porosity, and tissue mimicry for applications in regenerative medicine. It also improves nutrient diffusion and vascularization in 3D constructs, and reduces shear stress on cells via optimized bioinks, boosting viability higher than 85% [82].

Several deposition methods, such as continuous jet deposition, as well as tools offered by some of the available printers such as five-axis motion and in-flight curing, have been proposed for 3D microstructuring [83].

In the context of AJP, Seiti et al. tested three aqueous inks, AgNPs (commercial, diluted 1:4 for smoother structures), poly(3,4-ethylenedioxythiophene):poly(styrene sulfonate), PEDOT:PSS, (custom pseudo-elastic), and collagen-hydroxyapatite (Col-HAp, with/without glycerol), using strategies like continuous jet deposition (CJD), layer-upon-layer, and point-wise (PW), in this way achieving in under 10 min some 3D microstructures with heights of 200–590 μm, namely pillars with aspect ratio, AR, of 12 for AgNPs and AR = 4.5 for PEDOT:PSS, but also microcones with AR = 19 for Col-HAp [84]. Biocompatibility assays were pivotal: AgNPs showed high cytotoxicity (zero survival of human induced pluripotent stem cell (h-iPSC)-derived neural stem cells at 48 h), ruling out direct cell contact; PEDOT:PSS exhibited low toxicity (~20% reduced adenosine triphosphate, ATP, vs. plastic at 72 h, healthy fibroblast proliferation); and Col-HAp excelled, supporting confluent MC3T3-E1 osteoblast layers by day 7 with no dead cells in live–dead staining, mimicking bone composition for tissue engineering. Key biotechnological implications of these findings include rapid prototyping of conductive scaffolds for bioelectronics based on conducting polymers like PEDOT:PSS, or osteoinductive lattices made of composites like Col-Hap, while facing challenges such as ink pooling, parameter sensitivity (e.g., ShGF causing bending or process temperature), and overspray persist, all these aspects being crucial for repeatability in bioprinting applications through a layer-upon-layer approach.

Superhydrophobic surfaces (contact angle > 150°, sliding angle < 5°) also are increasingly exploited in biotechnology. The aim is to control aqueous solutions, biofluids, proteins, and cells without the need for closed channels or complex pumping systems [85]. Such surfaces are also being explored for DD and for modulating interactions with cells and tissues [86], as well as for their potential in preventing biofouling [87]. The goal of fabricating scalable, programmable superhydrophobic surfaces with controlled microstructures by means of AJP has been addressed by Zhong et al. [88], who utilized AJP to deposit polymer solutions of disulfide-polydimethylsiloxane in patterns upon exploiting solvent evaporation during droplet flight aimed to induce gelation and microgel formation. They found that solvents with high vapor pressures cause in-flight gelation, resulting in rough, superhydrophobic surfaces, while low-vapor-pressure solvents lead to smooth, less hydrophobic surfaces due to droplet coalescence. Additionally, heating can de-gel the polymer, allowing post-fabrication tuning of surface properties. These findings advanced the understanding of scalable methods for creating functional superhydrophobic surfaces with potential applications relevant to biotechnology, such as droplet manipulation, microreactors for reactant mixing, water–oil separation, and retardation of droplet evaporation.

### 4.1. AJP Aerosol Jet Printing for Surface Acoustic Wave Devices in Biotechnology

AJP has emerged as a transformative additive manufacturing technique also for the fabrication of surface acoustic wave (SAW) devices, addressing longstanding challenges in traditional lithographic methods that are labor-intensive, environmentally harmful, and reliant on cleanroom facilities [89]. The pivotal role of AJP in democratizing the access to high-performance SAW sensors lies in its maskless, direct-write capability, which enables rapid deposition of functional microstructures, such as silver interdigital transducers (IDTs) on lithium niobate (LiNbO3) substrates [90] using a wide range of conductive inks, including silver nanoparticles, silver nanowires, graphene, and PEDOT:PSS.

Key demonstrations underscore AJP’s versatility; sensors achieving operational frequencies of 40–87 MHz with tunable central frequencies via IDT finger/gap optimization have validated temperature sensing with coefficients closely matching theoretical predictions, offering low-cost alternatives for real-time monitoring [90]. In microfluidics, AJP streamlines SAW device production from multi-step, 40 h cleanroom processes to a single-step, 5 min fabrication, yielding acoustic performance comparable to conventional devices [91]. Optimized parameters (e.g., CGF/ShGF, atomization current, print speed for ~40 μm lines) have produced 20 MHz SAW thermometers with excellent linearity from 25–200 °C [92]. The SAW-AJP variant integrates SAW atomization for uniform adhesive films (1.5–5 μm, ~8% relative standard deviation, RSD, shear stress up to 550 kPa), aiding multilayer piezoelectrics [93].

AJP’s role extends profoundly into biotechnology, where SAW devices enable precise manipulation and sensing in complex biological environments. In acoustofluidics, AJP-fabricated SAW microfluidic platforms drive contactless particle/cell manipulation, such as acoustophoresis for size-based sorting of cells, exosomes, or biomolecules, bypassing biochemical labels and reducing biofouling risks, ideal for point-of-care diagnostics and organ-on-chip models. For biosensing, these sensors detect physical/chemical parameters (e.g., temperature, viscosity, biomarkers) in real-time via frequency shifts, with AJP enabling integration of biocompatible inks for wearable or implantable devices able to monitor the pH, glucose, or proteins in physiological fluids. In bioelectronics, SAW-AJP supports hybrid neuromorphic systems by printing conductive/shear-resistant films for flexible interfaces with biopolymers or organic electrochemical transistors, OECTs, facilitating brain–machine interfaces or prosthetic sensors with minimal invasiveness. Furthermore, rapid prototyping via AJP accelerates translation from bench to clinic, as seen in customizable LoCs for drug screening or pathogen detection [94], where reduced fabrication time enhances iterative design for personalized medicine. Future synergies with bioinks could yield fully printed SAW-biosensors for in vivo neuromorphic computing or tissue engineering scaffolds.

Despite advances, issues related to surface roughness, shorting, and frequency limits still persist, necessitating optimization for biocompatibility and long-term stability in biotic media.

### 4.2. AJP as an Enabling Technology for Microfluidics

So far, it has emerged that AJP combines a finer resolution, 3D/topographical capability, material efficiency in terms of no masks and minimal waste, and compatibility with a wide viscosity range and diverse inks, including polymers, proteins, nanoparticles, and piezoelectric materials. These features make AJP a powerful platform for LoC research, where fast iteration on device geometry and on-chip functionalization is crucial for applications such as diagnostics [95], biochemical assays [96], and particle or cell manipulation [97]. Indeed, in microfluidics, AJP has been used in two main ways. First, to print masters or molds for soft lithography, enabling rapid prototyping of PDMS devices with customized geometries such as steps, slopes, and complex channel networks, without photomasks or cleanroom processing [98]. Second, to locally deposit functional layers (e.g., hydrophilic polymers, biomolecules, conductive or piezoelectric inks) inside or on top of microfluidic channels, therefore enabling spatially resolved control of wettability [99], (bio)chemical reactivity [100], sensing elements [101], or acoustic actuation (as in SAW microfluidic devices [91]).

The article by Ćatić et al. explored a strategy for rapid prototyping of microfluidic devices combining complex geometries with precise, localized channel functionalization [98]. It showed that AJP could be used to fabricate high-resolution molds for soft lithography in an efficient and cost-effective way, yielding microfluidic channels with features down to 10 μm and enabling the selective deposition of functional coatings, such as hydrophilic poly(vinyl alcohol), PVA, at defined positions within the channels. The study highlighted that AJP supported versatile geometries, including steps and slopes, and allowed in-channel functionalization without compromising mold reusability, thus opening up new opportunities for flexible, customizable microfluidic device fabrication.

**Figure 2:**
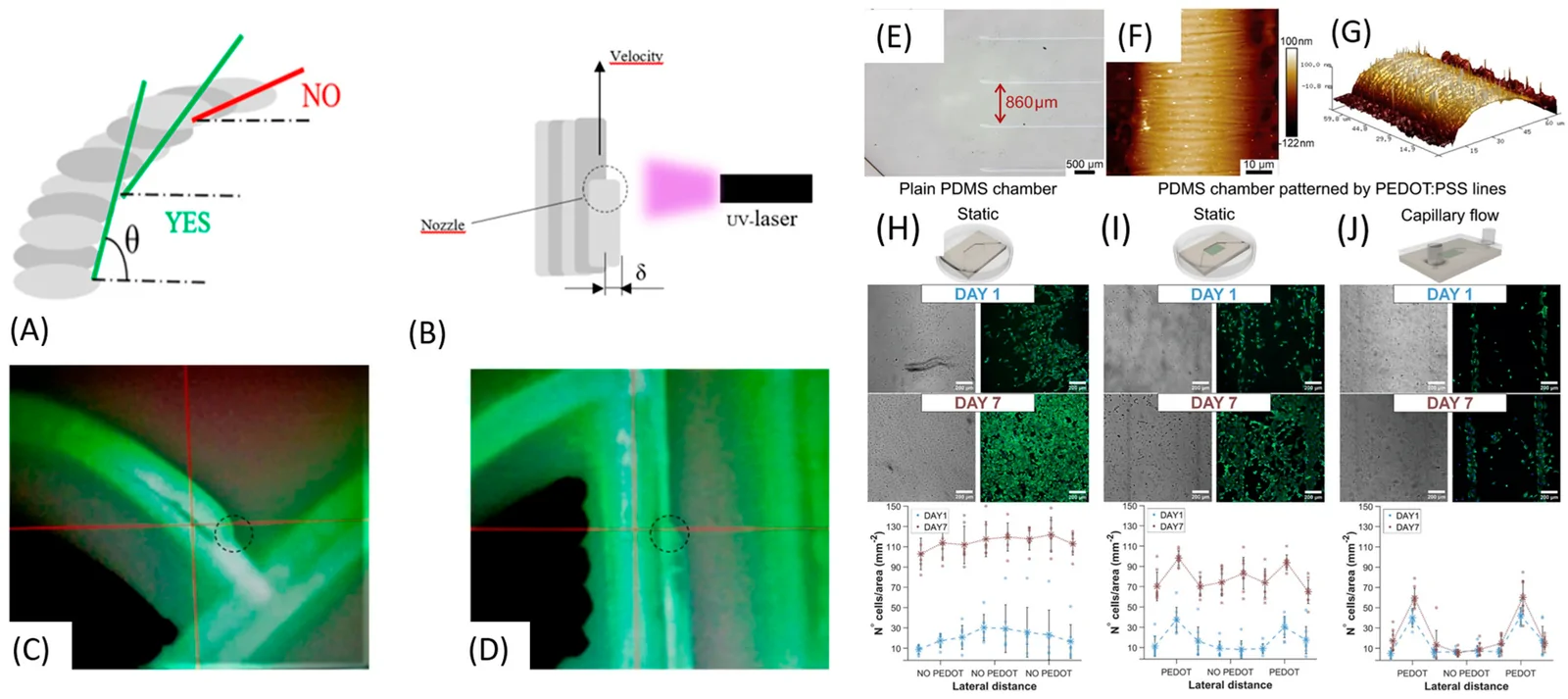
Microfluidic channel manufacturing by AJP onto electrodes for biosensing: (**A**) schematics of the concept of critical angle for optimal 3D printing by AJP in microfluidics and (**B**) representation of the line offset δ for stacked cantilevered lines allowing the implementation of stable out-of-plane structures; (**C**,**D**) process camera images acquired during the manufacturing of a microfluidic channel (adapted from [102]). Printed PEDOT:PSS parallel lines with (**E**) interline spacing < 1 mm (860 μm), (**F**) thickness ~100 nm, and (**G**) parabolic profile ~50 μm wide at base, ideal for single-cell accommodation while preserving fluid dynamics; EC culture and patterning on PEDOT:PSS-patterned PDMS. It is evidenced that (**H**) on Matrigel-coated PDMS, ECs distribute uniformly over the surface, while (**I**,**J**) on patterned substrates, ECs preferentially adhere and grow along PEDOT:PSS. Under capillary flow (**J**) ECs localize almost exclusively on the PEDOT:PSS lines. Bottom graphs report cell density per area (mean of 9 images from 3 devices, error bars: standard deviation), with eight regions analyzed per image; in patterned devices, the two regions containing PEDOT:PSS are labeled “PEDOT” on the lateral distance axis, and the remaining regions without polymer are labeled “NO PEDOT.” Scale bars: 200 µm; fluorescence: blue nuclei, green actin (adapted from [100]).

Microfluidics is the basis of the LoC concept, in which a single miniaturized chip integrates many functions typical of a conventional laboratory, such as sample preparation, chemical reactions, separations, detection, and analysis. AJP may support the realization of monolithically integrated LoC devices, from the substrate to the electronics and the microfluidic components themselves, within a rapid prototyping framework. In this context, the work by Di Novo et al. reported on a microfluidic electrochemical sensor platform entirely fabricated using AJP, providing advances in the understanding of AJP’s potential for streamlined, integrated biosensor manufacturing [102]. The authors addressed issues related to operator-dependent reagent deposition and microfluidic integration with miniaturized sensors. They demonstrated that AJP can produce microchannels made of NOA81, an ultraviolet (UV)-curable optical adhesive deposited to form cavities (Figure 2A–D) on previously printed electrode structures, with good repeatability and minimal variability, and validated the platform by detecting glucose within a clinically relevant range. These findings reveal that AJP enables rapid, support-free microfluidic device fabrication with consistent geometries, and that directly printed mediator and enzyme layers can produce effective glucose sensing, with a limit of detection around 2.4 mM. Moreover, Jing et al. developed low-cost, flexible, and conformable force sensors, suitable for biomedical and robotic applications, consisting of a novel microfluidic capacitive force sensor fabricated via AJP [101]. The sensor layout shared a microfluidic channel filled with a glycerol–water mixture and interdigitated electrodes on a flexible substrate. It exhibited a highly linear response to applied force up to about 9 N, with customizable performance characteristics (achievable by adjusting design parameters) in terms of tunable sensitivity and measurement range. The study demonstrated the sensor’s robustness over repeated cycles, its ability to conform to curved surfaces, and its potential for real-time force feedback, such as controlling a robotic clamp.

As mentioned above, cell manipulation is another prerogative of microfluidics, and guidance through microfluidic patterns is another key factor for different research lines in biotechnology, for instance, to form aligned fibers or layered structures, as well as networks that resemble native tissues [103]. Indeed, microfluidics allows the creation of in situ gradients to study migration of tumor or immune cells, to test inhibitors of these processes [104], and to implement tissue engineering, co-culture or organ-on-chip models [105]. The work by Mancinelli et al. explored the intersection of microfluidics and cell biology, focusing on how to better mimic the physiological conditions that influence endothelial cell (EC) behavior [100]. This has been done with an overall experimental approach involving AJP to create precise patterns of PEDOT:PSS (line width 50 µm, thickness 100 nm) within microfluidic chambers (Figure 2E–G). The authors assessed the biocompatibility of the PEDOT:PSS patterns, their ability to support ECs adhesion, and the effectiveness of the patterns in promoting cell alignment under capillary flow conditions, while under static conditions the cells proliferated across the entire surface (Figure 2H–J).

The as-discussed examples are indications of how AJP allows eliminating the cleanroom PDMS casting needed in traditional microfluidics in order to (i) form 3D freeform channels, (ii) promote embedded integration of functional layers or (bio)electronic devices, and (iii) offer a room-temperature processing ideal for biologics, all this in a scalable and cost-effective manner. The current limitations of AJP in resolution (i.e., overspray and voids) and gas set-up complexity for reliable scalability, both require mitigation approaches in ink formulation and gas line features that are, at present, already faced and discussed in the literature [99].

### 4.3. AJP at the Frontier of Neuroprosthetics

**Figure 3:**
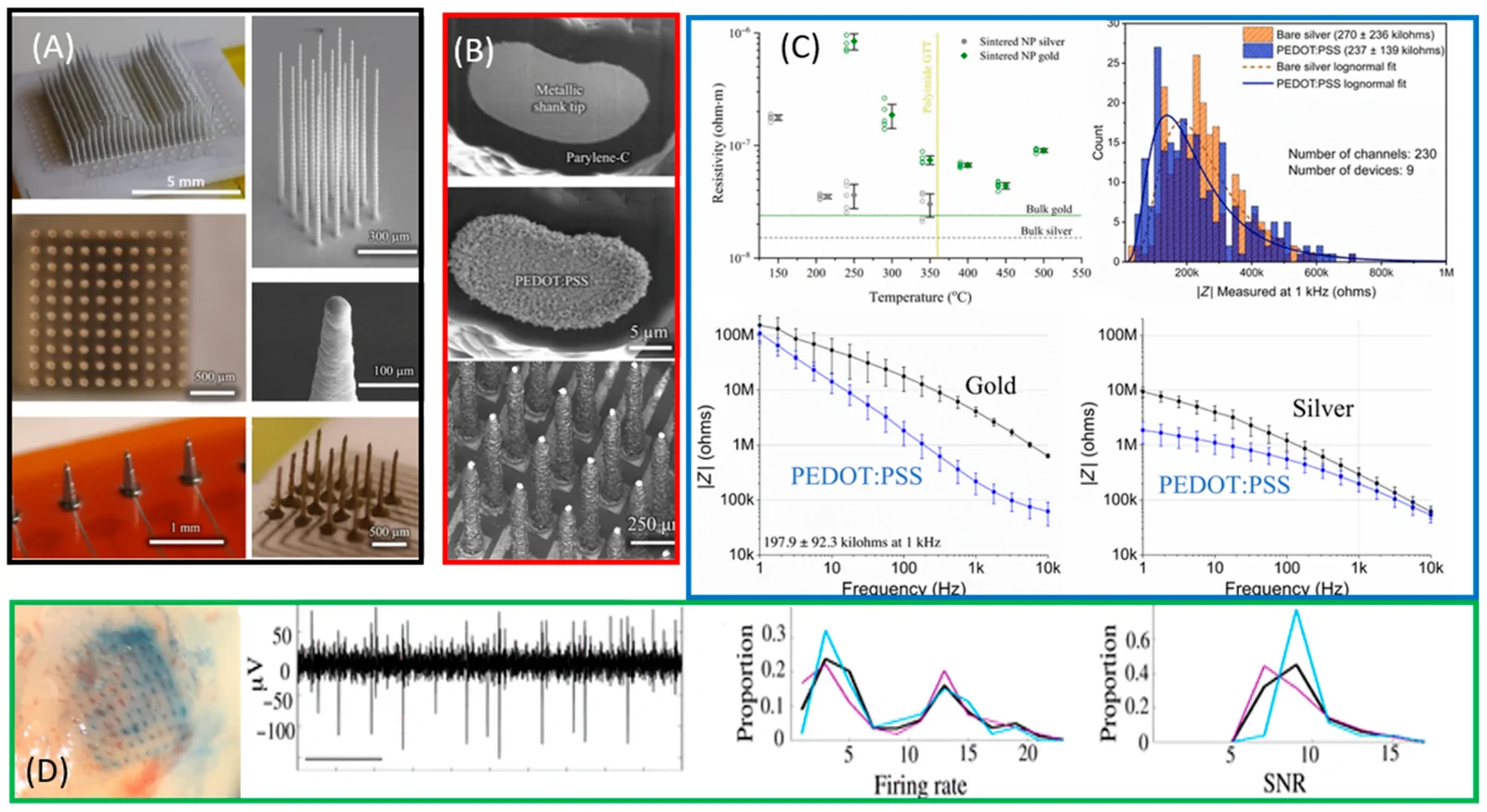
(**A**) Ag shank arrays with different pitches AJ-nanoprinted on various substrates; (**B**) parylene C and PEDOT:PSS deposition on a Ag tip of the array; (**C**) electrical properties and impedance of bare and PEDOT:PSS-coated Ag array (by comparison with nominal gold ones); (**D**) trace of the implantation of 32-channel electrode on rat brain and representative of the brain activity from a single channel, together with average firing rate and SNR of neurons from three different channels (adapted from [111]).

The literature reports several specific examples of applications in neuroprosthetics, describing technologies directly interacting with the nervous system (whether peripheral nerves, the spinal cord, or the brain) by delivering electrical stimulation aimed at managing various medical conditions. Examples include devices like cochlear implants, cardiac pacemakers, and brain–computer interfaces [106,107,108]. Despite the currently limited use of AJP in other biotechnology sectors, this technique finds large employment in such areas of applied research. In particular, studies at the frontier with bioelectronics describing different types of electrode platforms for neural recording and stimulation may be found. Kim et al., for instance, printed platinum-based microelectrodes on flexible polyimide substrates via AJP, demonstrating probes with enhanced signal resolution, durability, and minimal tissue damage for weak neural signal detection [109], while Dijk et al. demonstrated PEDOT:PSS-coated platinum electrodes for deep brain stimulation, showing improved charge transfer and reduced impedance, aiding outcomes for Parkinson’s disease treatment [110]. Finer applications are found in the work by Saleh et al., which showed the high-resolution printing of a customizable neural probe consisting of Ag microelectrode array (CMU Array) shanks, specifically fabricated via AJP on customizable substrates (alumina, Kapton, printed circuit board, PCB) and achieving unprecedented densities up to 2600 shanks/cm^2^ (200 µm pitch) with variable lengths between 0.1–3 mm and tip diameters of 10–150 µm (Figure 3A).

Shanks and multilayer routing were coated with PEDOT:PSS (Figure 3B) for impedance reduction (~200 kΩ at 1 kHz, Figure 3C) and parylene-C insulation, yielding robust, flexible probes with more than 98% yield and buckling forces higher than 80× insertion force in agarose brain phantom. In vivo mouse cortex recordings (Figure 3D) demonstrated single-unit isolation (signal-to-noise ratio, SNR 8.6, firing rates ~9 Hz), minimal tissue damage, and fast prototyping, surpassing silicon Utah arrays in customization, density, and cost. This platform enables study-specific neural targeting across brain volumes for neuroscience and brain–computer interfaces [111].

In addition, Park et al. developed ultra-thin, stretchable electrode arrays for spinal cord stimulation; their nerve-adhesive design adapted to brain/spinal tissues without mechanical stress, maintaining stable neural recording/stimulation [112].

It is worth noting that all the aforementioned studies are quite recent, being published within the past five years. This reflects the fertile ground that AJP-based electrode fabrication, long established since AJP emerged as a paradigm for 3D-printed electronics, now finds in the new exploratory phase driven by advances in the Internet of Things (IoT), personalized medicine, and brain–computer interfaces. On the applications side in the context of cell guidance engineering, which focuses on the design and development of advanced surfaces and architectures capable of guiding cells and tissues via cell adhesion, proliferation, and migration, Capel et al. explored AJP for the fabrication of micro-scale conductive PEDOT:PSS patterns to direct neuronal growth. By optimizing ink composition (1.04% PEDOT:PSS, 20% ethylene glycol, 1% 3-glycidoxypropyltrimethoxysilane, GOPS) and printing parameters, the authors achieved uniform features with widths ranging from 15 to 50 µm and surface roughness below 3.5 nm. The patterned substrates significantly enhanced SH-SY5Y cell alignment, particularly on narrower tracks (~20 µm), and the addition of a poly(potassium 3-sulfopropyl methacrylate) (PKSPMA) polymer brush further improved cellular organization by introducing cell-repellent regions. Notably, complex patterns were produced rapidly (within 6–20 s), demonstrating AJP’s capability for high-resolution, fast prototyping of customizable neural guidance platforms [23].

In neuroprosthetics, cell guidance opens perspectives towards neural tissue engineering, which is targeted in the study of solutions for repairing, regenerating, or replacing tissues of both the central and peripheral nervous system. It impacts on peripheral nerve regeneration and spinal cord repair, on in vitro neural network models (brain-on-chip) and bioelectronic neural interfaces, as well as on 3D-printed neural networks and soft implantable neuroprostheses. The potential of AJP in cell guidance for neural tissue engineering has been studied by Seity et al. [113], who demonstrated bioelectronic interfaces on micropatterned substrates for neural tissue engineering made of printed PEDOT:PSS. The authors optimize key process parameters, including focusing ratio (~2), platen temperature (~40 °C), and carrier gas flow (~40 sccm), enabling the deposition of ~33 μm thick, ~2 mm wide electrodes with low resistance (~16 Ω) and impedance values of 1–2 KΩ at 1 kHz in saline. The approach was successfully transferred to Parylene-C-coated silicon scaffolds, preserving line morphology and electrical performance, although residual diethylene glycol in the ink leads to dose-dependent cytotoxicity.

Some of the applications reported here show that the AJP technique can be proposed as a valid alternative to MEMS techniques in the fabrication of biosensors. Compared with conventionally fabricated biosensors, AJP biosensors offer significant advantages in terms of rapid prototyping, maskless fabrication, material efficiency, and direct printing on flexible, curved, or non-planar substrates. These characteristics facilitate the development of customized sensing platforms and accelerate design iteration. In terms of analytical performance, several proof-of-concept studies have demonstrated sensitivities and detection limits comparable to those achieved by conventionally fabricated devices, particularly for electrochemical and transistor-based biosensors. However, conventional microfabrication techniques still provide superior process standardization, batch-to-batch reproducibility, and industrial scalability. Furthermore, long-term stability and large-scale validation of AJP-fabricated biosensors remain less extensively investigated. Therefore, while AJP represents a highly promising manufacturing approach for next-generation biosensing devices, further studies are required to establish its reproducibility and reliability under clinically relevant operating conditions.

### 4.4. Strategies for Drug Delivery and Tissue Engineering

AJP versatility (as per capability of integrating structural, electrical, and biochemical functionalities within a single platform) translates into the ability to engineer microstructured systems, tailor surface properties, and precisely control drug loading and release profiles, opening new opportunities for personalized therapies and multifunctional biomedical devices in DD systems, from platforms (e.g., microneedle, MN, arrays) to formulations.

MNs are a well-established and widely studied system for transdermal DD, whose implementation is primarily carried out by 3D printing methods. Microneedles are 3D-printed by using, for instance, stereolithography [114], 3D printing based on digital light processing [115] and two-photon polymerization [116]. To guarantee a proper penetration of the stratum corneum (100–150 µm thick), sharp MNs should be 400–600 µm in height (tip of 10–20 µm), with an aspect ratio of 3–4 (in plane dimension of 100–150 µm for shape stability) [117,118]. Taking into account that MN density in dedicated therapy-efficient arrays ranges between 100 and 1000 MN/cm^2^ [119], with its finer resolution AJP emerges as the optimal technique for easy MN integration in well-operating patches. Furthermore, established 3D methods for MN array implementation are based on mechanically resilient polymeric materials, while DIW also permits the deposition of metallic inks and organic blends/compounds in the form of viscous inks, with objective advantages promoted by enhanced functionalization capabilities and electrode operation useful for biosignaling and biosensing applications.

The work by Zips et al. has pursued a hybrid IJP/AJP approach to fabricate MNs using a conductive PEDOT:PSS/multi-walled carbon nanotube (MWCNT) composite ink, achieving conductivities on the order of ~10^2^ S·m^−1^. The resulting MNs exhibited well-defined micro-scale geometries (≈10 μm diameter, ≈30 μm height) and the capability of partially penetrating dielectric layers, enabling vertical electrode architectures that have been tested in vitro for recording extracellular signals from HL-1 cardiomyocyte-like cells, confirming both biocompatibility and functional performance [38].

A recent work by Ako et al. has demonstrated the successful use of an organic compound, namely a polyvinylpyrrolidone (PVP)-trehalose system, to produce microneedles with adequate mechanical strength and rapid dissolution characteristics [120]. Systematic optimization of key process parameters, including atomizer flow rate, stage temperature, print speed, and focusing ratio, was essential to achieving high-quality structures. Optimized conditions enabled the fabrication of microneedles exceeding 500 µm in height, with sharp geometries and consistent morphology. Importantly, the printed microneedles exhibited effective penetration into porcine skin models, confirming their functional capability. Physicochemical analyses indicated that the mild printing conditions preserved material integrity, supporting the potential incorporation of sensitive therapeutics. This work promotes AJP as a flexible and scalable approach for efficient microneedle fabrication, offering advantages in material compatibility and structural control.

Recent efforts have further extended AJP towards functional tissue engineering approaches and pharmaceutical formulations, demonstrating its potential to assist tissue repair and to precisely control drug solid states and enhance dissolution performance, respectively.

Although based on extrusion printing rather than AJP, the work by Ganguly and Tang illustrates how ink formulation and post-printing treatment can be combined to engineer sustainable biopolymer-based scaffolds. By optimizing a cellulose acetate/cellulose nanocrystal ink and applying solvent-exchange post-processing, the authors obtained lightweight porous constructs with improved mechanical and thermal properties. These findings highlight the importance of considering printing and post-processing as an integrated workflow for controlling scaffold porosity and performance, an approach that may also support the development of AJP-compatible biomaterial systems [121].

Aerosolization of nanoparticle surfaces represents a valid and smart approach to impart a desirable multifunctionality to systems for tissue engineering, such as 3D scaffolds, allowing independent control of surface chemistry to assist cell expression, nano-roughness for promoting their proliferation, and synergistically implementing an active molecule release, for eventual treatment, on implantable devices. AJP systems may be easily forced towards a spray-coating regime, for instance by inducing turbulence in the aerodynamic sheath gas flow [36]. In this context, the work by Patelli et al. is particularly significant, as it provides insights into the use of AJP for surface control combined with release functionalities in implantable devices [122]. Herein, a two-step process combining aerosol deposition of nanoparticles with atmospheric pressure plasma jet (APPJ) coating, enabling precise tuning of submicron roughness, surface functional groups (amine or carboxyl), and drug release capabilities, even on temperature-sensitive polymers like polycaprolactone (PCL, a synthetic biopolymer widely used for scaffold manufacturing), has been developed. The method has been demonstrated to effectively enhance cell adhesion and proliferation (by about 20% on titanium and 100% on PCL) while also demonstrating the controlled release of embedded fluorophores.

**Figure 4:**
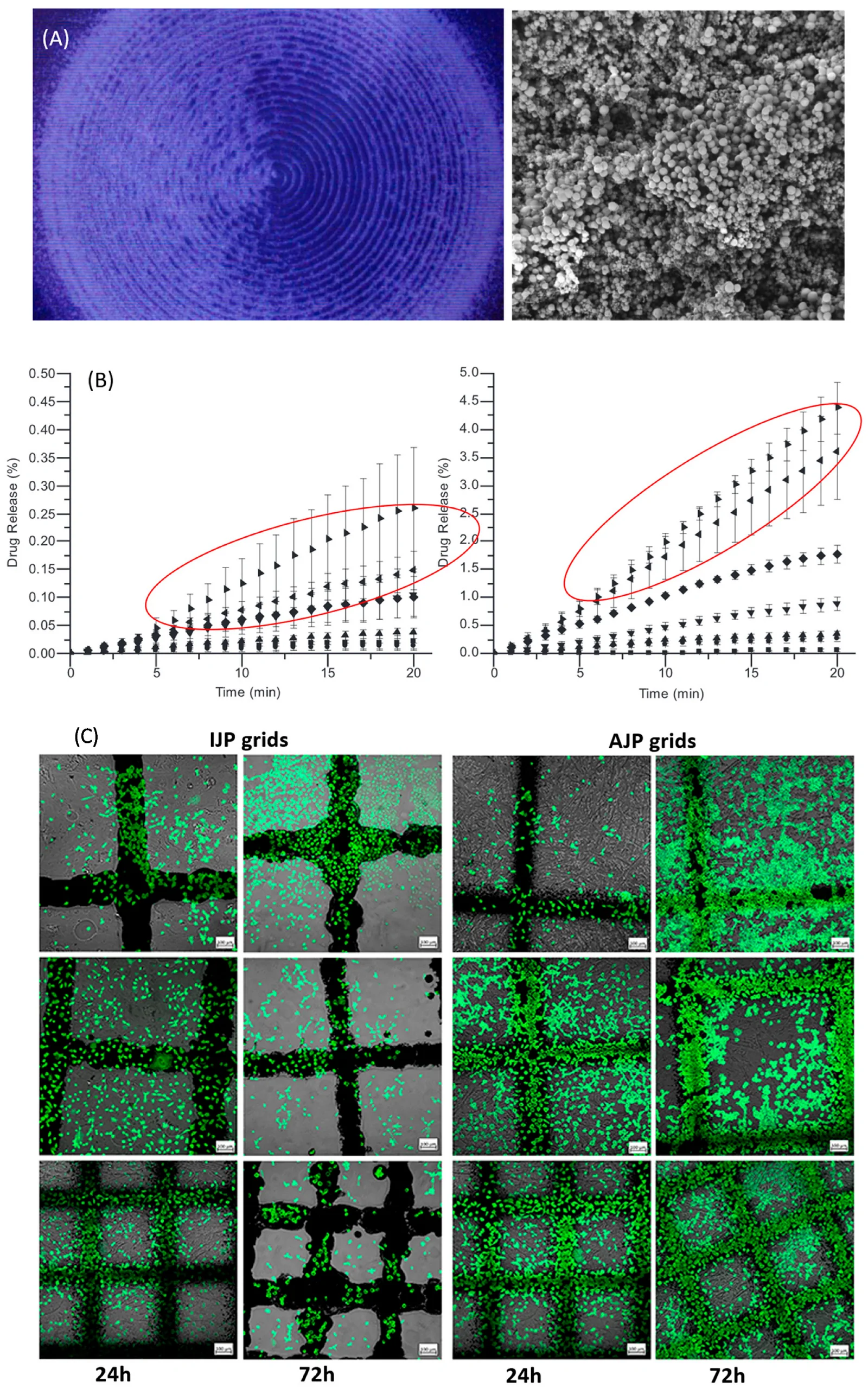
(**A**) Image and SEM of printed fenofibrate-PVP K30 with 75 and 80% (*w*/*w*) of K30 content, respectively; (**B**) percentage of drug release from compacts (left) and printed samples (right) of fenoblate-PVP K30; red circled regions are at 75–80% of K30 content (adapted from [52]); (**C**) morphological staining of L929 fibroblasts cultured on patterned substrates patterned with IJP (**left**) and AJP (**right**), analyzed at 24 h and 72 h, using grid geometry, demonstrating superior performance by AJP patterns (adapted from [37]).

Finally, the work by Turner et al. [52] investigates AJP as a high-resolution additive manufacturing technique to produce amorphous solid dispersions of fenofibrate (a poorly soluble drug classified as class II within the Biopharmaceutics Classification System). By formulating drug–polymer inks based on poly-vinylpyrrolidone K30 (PVP K30) with high polymer content (≥75% of PVP K30), the authors achieved fully amorphous systems (Figure 4A) with significantly enhanced dissolution, up to ~10× versus physical mixtures, Figure 4B.

Hence, AJP enables precise control over drug distribution, morphology, and dose via layer-by-layer deposition, without any thermally induced stress effect. The printed formulations indeed showed good stability and reproducibility, highlighting AJP as a promising platform for tailored drug delivery, particularly in personalized and small-scale pharmaceutical applications.

The current literature, although limited, also evidences the potential of AJP as a tool for tissue engineering. More broadly, recent advances in dental and craniofacial tissue engineering show that 3D-printed scaffolds can provide patient-specific architectures and controlled microenvironments for tissue regeneration, while highlighting surface biofunctionalization, long-term biocompatibility, and immune response as critical requirements for clinical translation [123]. Such potential emerges, in particular, from significant examples that well highlight the versatility of this technique. Indeed, AJP has been shown to play a central role in the development of tissue engineering, being accordingly used in tissue regeneration applications to implement the manufacturing of freeform alginate hydrogels enriched with cells for scaffolds manufacturing [124], and to fabricate conducting patches for cardiac tissue engineering [125]. The first example indeed showed an encapsulation strategy for a cell-dispensing method relying on an aerosol-spraying process for fabricating alginate scaffolds enriched with preosteoblast (MC3T3-E1) cells. Herein, the authors present a modified solid freeform fabrication approach to generate thick, porous, cell-laden alginate scaffolds with controlled internal architecture. By combining extrusion-based deposition with aerosolized CaCl_2_ cross-linking, the method enables localized and immediate surface gelation during layer-by-layer assembly. Systematic optimization of key parameters, including nozzle diameter, aerosol flow rate, and CaCl_2_ concentration, resulted in scaffolds with highly interconnected pores and favorable mechanical stability. Importantly, high initial cell viability (~86%) was achieved and sustained during long-term culture, supporting cellular proliferation and distribution. These findings demonstrate that controlled aerosol cross-linking significantly enhances structural integrity and biological performance, offering a promising strategy for fabricating clinically relevant hydrogel constructs for tissue engineering applications.

The second work well addresses the control of printing features and functional characteristics offered by innovative inks, whose combination is beneficial for tissue repair applications. Aimed at addressing key limitations in cardiac tissue engineering, namely insufficient electrical conductivity and lack of structural anisotropy, the study shows the development of electrically conductive, 3D-printed cardiac patches using Ti_3_C_2_T_x_ MXene nanosheets patterned onto polyethylene glycol (PEG) hydrogels via AJP. The experimental results demonstrated that, while MXenes enable high-resolution patterning, their incorporation in PEG hydrogels enhances cardiomyocyte viability, alignment, and maturation, as evidenced by increased expression of cardiac markers and improved electrophysiological performance, including faster conduction and synchronized beating.

Printed structures aiding cell guidance, an approach already discussed in the case of neuroprosthesis, have been demonstrated by Sosnowicz et al. and addressed as promising for tissue engineering applications, where precise control of cell–material interactions is required [37]. They investigated the use of graphene nanoplatelet micropatterns fabricated on flexible polymer substrates to regulate cell behavior, with particular attention to the influence of printing techniques on biological performance. The authors compared IJP and AJP, observing that both methods produced stable and cytocompatible graphene patterns. However, they reported significant differences in microstructure, morphology, and surface properties between the two approaches. In particular, they found that AJP-generated patterns exhibited higher roughness, porosity, and more pronounced three-dimensional features, which promoted enhanced fibroblast adhesion, proliferation, alignment, and guided migration, while IJP patterns were smoother and less effective in directing cell behavior (Figure 4C).

This example provides further evidence that AJP, whether within a direct manufacturing [125] or a complementary coating process using AJP as a coating technique [37,124], can be effectively employed in the development of biotechnological systems, demonstrating efficiency in DD applications and enhanced performance relative to IJP in the field of tissue engineering. It is worth mentioning that, to date, there is a lack of literature examples going beyond the mere demonstration of AJP potential in DD and tissue engineering. However, in light of the advantages offered by this technique, further research should place greater emphasis on its use, aiming to demonstrate its suitability in the context of advanced biological experiments.

## 5. Discussion

The reviewed literature demonstrates that AJP is an emerging and versatile platform for biotechnology, enabling applications ranging from biosensing and microfluidics to tissue engineering and drug delivery. Its main strengths lie in its high resolution, broad ink compatibility, and ability to process complex biomaterials, allowing precise control over both geometry and functionality.

A key aspect is the strong interplay between printing parameters, material formulation, and biological response. Features such as surface roughness, porosity, and microstructure, directly influenced by the AJP process, have been shown to regulate cell adhesion, proliferation, and alignment, also demonstrating superior performance with respect to IJP counterparts. At the same time, AJP enables the fabrication of multifunctional systems through the integration of structural, electrical, and biochemical cues, which is particularly relevant for advanced platforms such as bioelectronics and tissue-engineered scaffolds. In this context, insights from 3D-printed electronics are instructive: AJP currently acts mainly as a complementary technique rather than a fully in-line fabrication method, due to the need for optimized ink formulations and the difficulty in achieving uniform, high-quality features comparable to conventional processes. As a result, its role is more effectively framed within hybrid integration strategies, where AJP is combined with standard fabrication techniques whenever high performance and reliability are required.

In biotechnology, this limitation may be less restrictive. Hence, AJP could in principle support more integrated, in-line prototyping approaches. At present, consolidated applications of AJP may involve coating processes or the integration of printed electronics onto biomimetic systems, including neuromorphic interfaces and tissue engineering platforms. However, the fabrication of true bulk 3D structures remains challenging, as the out-of-plane thickness achieved per deposition step is still limited (a single pass ranging from hundreds of nanometers for common polymers [15] to even tens of micrometers [126]), often requiring multiple processing cycles.

Although the fabrication of bulk 3D structures remains inherently more challenging than planar patterning, the limited thickness achieved in a single deposition step does not represent an insurmountable constraint. Several studies have demonstrated that the vertical growth of aerosol-jet-printed architectures can be significantly enhanced through a suitable optimization of process parameters, including carrier and sheath gas flows, deposition speed, substrate temperature, and ink rheology. In particular, the use of highly viscous formulations, reduced stage velocities, and printing conditions promoting increased material accumulation per unit area can favor layer stacking and the generation of structures with appreciable out-of-plane dimensions. Furthermore, AJP can be intentionally operated in wider-beam or spray-like deposition regimes, trading lateral resolution for higher deposition rates and greater material build-up. Such approaches may be especially advantageous for the fabrication of porous scaffolds and tissue-engineering constructs, where large surface area, interconnected architectures, and volumetric growth are often more important than micrometric feature resolution. It should also be noted that the need for multiple deposition passes is not unique to AJP but rather represents a common limitation of most additive manufacturing technologies based on liquid inks, where three-dimensional constructs are obtained through sequential material accumulation. However, AJP still remains the most promising method for fine patterning by approaches based on liquid precursors, useful for several applications in biotechnology, ranging from cell guidance to printed microfluidics.

Nevertheless, the wide viscosity range of printable inks enables the use of polymeric systems with tunable chain length and mechanical properties, offering opportunities for engineering structurally and functionally complex architectures. This aspect is particularly relevant for next-generation biofabrication approaches, including flexible, hybrid, and 4D systems capable of dynamic responses such as shape change [33]. In this context, strategies reported in the literature to tailor material properties are particularly relevant, as they provide effective tools for the development of advanced composites that combine multiple functionalities, including bioreactivity, environmental stability, structural performance, and solution processability. Examples of AJP-printable composites include multifunctional gelatin-based polymer matrices capable of regulating cellular proliferation [127], photocurable resins in combination with gelatins/hydrogels designed for the fabrication of 3D-printed scaffolds [128] and various polysaccharides [129].

Despite these promising features, several challenges remain, including overspray effects, bioink stability during atomization, and the need for formulations that simultaneously ensure printability and biological functionality. Moreover, most studies are still limited to proof-of-concept demonstrations, with limited validation in long-term or in vivo conditions. Although AJP clearly shows strong potential as an enabling technology in biotechnology, further efforts are required to consolidate its role through systematic process optimization, advanced ink design, and validation in complex biological environments.

A critical aspect for the adoption of AJP in biotechnology concerns the effects of atomization and droplet delivery on the structural integrity and biological functionality of printed materials. Evidence is heterogeneous, as it does not indicate that aerosolization is either intrinsically damaging or entirely harmless. Instead, biological activity depends on atomization mechanism, biomolecule properties, bioink formulation, exposure and recirculation time, gas flows, nozzle geometry, and post-deposition treatment.

For proteins and enzymes, biological activity can be retained under appropriate processing conditions. Bovine serum albumin and horseradish peroxidase have been successfully deposited, with the latter retaining enzymatic activity after printing and mild thermal treatment [55]. Ultrasonic AJP has also preserved the functionality of bovine serum albumin, streptavidin, and anti-carcinoembryonic antigen antibodies, enabling well-performing immunoassays [56]. Thus, ultrasonic atomization is not necessarily incompatible with protein-based inks. Nevertheless, retained function does not prove complete structural preservation, which may vary with molecular architecture, formulation, residence time, and analytical endpoint. As regarding the process temperature, excessive temperatures may promote protein unfolding, aggregation, loss of enzymatic or recognition activity, and accelerated solvent evaporation, potentially altering both ink rheology and deposition quality. Conversely, excessively low temperatures may increase viscosity, reduce aerosol-generation efficiency, or induce gelation in temperature-sensitive formulations. Post-deposition heating should therefore be minimized and applied only within a protein-specific validated temperature range.

Collagen-based inks provide more heterogeneous findings. Collagen type II appeared less susceptible to process-induced alteration than collagen type I [79]. Partial denaturation has also been observed in recombinant human collagen type III [80]. Conversely, ultrasonic printing caused no notable denaturation in other collagen formulations and improved molecular alignment and surface uniformity [81]. Furthermore, collagen degradation following ultrasonic atomization has been observed in collagen-hydroxyapatite formulations [53]. Hence, collagen susceptibility also depends on type, source, formulation, exposure time, printing conditions, and analytical method. Similarly, DNA integrity depends on molecular size and atomization mechanism. High-molecular-weight DNA may be damaged during ultrasonic atomization, even if it has been reported that DNA is better preserved using pneumatic atomization [55], whereas other DNA formulations maintained biological functionality following ultrasonic delivery [56]. This discrepancy may reflect differences in molecular weight, formulation, exposure, and analytical endpoints. Pneumatic atomization may therefore be preferable for long or mechanically sensitive nucleic acids, while ultrasonic atomization may suit shorter or appropriately formulated DNA.

From a mechanistic perspective, sensitive biomolecules may be affected by shear and extensional stresses, cavitation, local temperature variations, and repeated recirculation within the atomizer. Ultrasonic and pneumatic atomization should be considered separately because they create different mechanical environments. Compared with inkjet printing, where substantial stresses may occur during droplet ejection, AJP separates aerosol generation from transport and aerodynamic focusing through a sheath gas. The non-contact deposition event is relatively gentle, whereas aerosol generation, recirculation, and transport may be more critical. However, AJP is not necessarily less damaging than inkjet printing. Its biocompatibility should be evaluated by considering the complete printing workflow rather than only the final deposition step [130]. Similar considerations apply to cell-containing bioinks, although few studies have directly investigated the aerosolization of living cells by conventional AJP. Cell viability may be influenced by droplet size, impact velocity, operating pressure, nozzle diameter, and culture conditions. High operating pressures and small nozzle diameters have been associated with reduced viability in jetting-based bioprinting [131]. Direct atomization-induced damage must, however, be distinguished from material-related biological responses. The cytotoxicity of silver nanoparticles, cellulose nanocrystals, conductive additives, or residual solvents, for example, should not be directly attributed to aerosol generation [84].

Approximately 86% cell viability in alginate scaffolds fabricated using aerosolized CaCl_2_ indicates compatibility with cell-laden constructs [124]. However, the cells were incorporated into the extruded alginate phase and were not directly aerosolized. This supports aerosol-assisted cross-linking of cell-containing scaffolds, but not the safety of direct cell aerosolization.

Overall, preservation of biological function should be regarded as an application-specific process-optimization challenge rather than an intrinsic limitation of AJP. Future studies should report atomization mechanism, residence time, gas flows, nozzle dimensions, temperature, and bioink rheology, together with quantitative measurements of molecular integrity, biological activity, and cell viability before and after printing. Direct comparisons between ultrasonic and pneumatic atomization using identical formulations and standardized endpoints are particularly needed to define reliable processing windows for protein, collagen, nucleic acid, and cell-containing bioinks.

As regards the reliability of artifacts printed by AJP systems, it is worth noting that the use of AJP is affected by the same detrimental factors encountered in other printing technologies, while the translation of these approaches into clinical practice is more closely related to the performance of the devices and materials with respect to the intended application than to the fabrication technique itself. In this regard, AJP presents both advantages and disadvantages compared with more widely used 3D printing technologies. The key to achieving reliable applications lies in the combined use of different manufacturing techniques, each offering specific strengths and superior features for particular fabrication requirements.

To provide a broader perspective beyond bioink formulation, Table 2 summarizes the principal advantages and limitations of AJP across the main biotechnological application fields discussed in this review. The table highlights that the strengths of AJP are generally associated with its high-resolution, maskless, and multimaterial capabilities, whereas its limitations mainly arise from throughput, atomization-related effects, and the difficulty of rapidly building large three-dimensional volumes.

Overall, our analysis suggests that AJP should not be viewed as a universal replacement for existing biofabrication techniques. Rather, its greatest impact is expected in hybrid manufacturing approaches, where its unique capabilities in high-resolution patterning, surface functionalization, and multimaterial integration complement the strengths of extrusion-based, lithographic, or molding techniques.

From a manufacturing perspective, the development of standardized and Good Manufacturing Practice (GMP)-compliant workflows is essential to ensure reproducibility, batch-to-batch consistency, and quality control. Additional efforts are also required to establish validated sterilization protocols, as conventional sterilization methods may alter the physicochemical properties of printed biomaterials or affect the performance of biological components. Long-term storage stability represents another critical issue, particularly for formulations containing proteins, nucleic acids, cells, or other bioactive agents. Comprehensive studies on shelf-life, functional preservation, and storage conditions are still limited. Furthermore, although encouraging in vitro results have been reported, extensive in vivo investigations are needed to assess biocompatibility, immune responses, toxicity, and long-term functionality of AJP-fabricated biomedical devices and constructs.

Finally, regulatory approval pathways remain largely unexplored for many AJP-enabled technologies. Close collaboration among materials scientists, bioengineers, clinicians, and regulatory bodies will be crucial to establish standardized testing protocols and accelerate the translation of AJP-based solutions from laboratory research to real-world biomedical and healthcare applications.

As a final remark, similar to what is happening in many research fields, 3D printing routes, including AJP, are currently taking advantage of the powerful support of Artificial Intelligence (AI) tools. Machine Learning (ML) approaches have met AJP and DIW approaches during the last decade, shedding light on process optimal output, which is strongly influenced by complex interactions among process parameters. Through several ML approaches, for instance by combining experimental sampling, clustering, classification, and transfer learning [132], as well as by using knowledge transfer techniques [133], improved control over printed line morphology has been demonstrated and validated through case studies. Predicting process–structure relationships in bioprinting, as done for instance in the case of structures deposited from filaments by Fused Deposition Modeling [134], as well as mechanical/morphological properties and cell behavior/biological performance of functional surfaces fabricated and/or treated by 3D printing routes, similarly can further ameliorate the prototyping of biotechnological tools when using the material-wise highly versatile AJP method.

## 6. Conclusions

Aerosol jet printing is emerging as a versatile enabling technology for biotechnology, combining maskless digital fabrication, high spatial resolution, broad material compatibility, and conformal deposition on planar and non-planar substrates. The studies examined in this review demonstrate its applicability to surface biofunctionalization, biosensing, microfluidics, cell guidance, tissue engineering, drug delivery, and bioelectronic and neuroprosthetic interfaces. In these fields, AJP enables not only the deposition of biological and functional materials but also the control of geometry, surface morphology, porosity, roughness, and the spatial distribution of biochemical and electrical cues. These capabilities are particularly relevant when cell adhesion, proliferation, migration, alignment, or biomolecular recognition must be controlled at the microscale.

The suitability of AJP for biotechnology, however, cannot be evaluated independently of ink formulation and processing conditions. Biological performance arises from the interplay among material rheology, atomization mechanism, droplet characteristics, gas flows, nozzle geometry, working distance, substrate properties, deposition speed, and post-processing conditions. The preservation of protein structure, nucleic acid integrity, enzymatic activity, and cell viability must therefore be assessed for each material and intended application, rather than regarded as an intrinsic and universally predictable feature of the technique. The heterogeneous results reported for protein- and collagen-based formulations reinforce the need for systematic correlations between processing conditions and structural, molecular, and biological outcomes.

Atomization represents one of the most critical process stages. Although aerodynamic focusing and non-contact deposition may provide relatively mild conditions during material delivery, ultrasonic or pneumatic aerosol generation can affect shear-sensitive biomolecules and cell-containing formulations. The magnitude of these effects depends on the biological component, ink properties, atomization mechanism, and selected processing window. Atomization should therefore be regarded not as an absolute barrier to biotechnology applications, but as an optimization challenge requiring the appropriate selection of ink composition, printing parameters, atomization method, and substrate conditions. A further limitation concerns the fabrication of bulk three-dimensional structures. AJP can produce multilayer architectures, pillars, lattices, coatings, and high-aspect-ratio elements, but the limited amount of material deposited during each pass may increase the processing time required to obtain macroscopic volumes. Vertical growth can be enhanced by tuning ink viscosity, deposition speed, gas flows, substrate temperature, and material accumulation per unit area. Wider-beam or spray-like regimes, including the use of dedicated wide-deposition heads, may be employed when productivity and volumetric build-up are more important than lateral resolution. Nevertheless, the trade-off among resolution, productivity, structural fidelity, and biological performance remains important, particularly in scaffold fabrication. Multiple-pass deposition is also common to most additive manufacturing techniques based on liquid inks and is not an exclusive limitation of AJP. Additional challenges include overspray, variations in feature morphology, narrow processing windows for certain biomaterials, and process reproducibility. Most studies remain at the proof-of-concept stage. Technological maturation therefore requires functional assessments under conditions that progressively reproduce the intended application, including long-term stability, retention of bioactivity, interactions with complex biological environments, and performance of the complete material–device system. For biomedical translation, particular attention should also be given to sterilization, storage stability, manufacturing consistency, quality control, and regulatory requirements.

Despite the remarkable progress achieved in the application of AJP to biotechnology and biomedical engineering, several challenges must still be addressed before widespread clinical translation can be realized. Most reported studies remain at the proof-of-concept stage, with limited validation under physiologically and clinically relevant conditions. The translational potential of AJP will ultimately depend more on the performance, safety, stability, and reproducibility of the resulting material–device system than on the printing technique alone. AJP should therefore not be considered a universal replacement for established fabrication methods, but rather a valuable component of hybrid manufacturing workflows. Conventional or extrusion-based techniques may provide bulk structural components, while AJP can introduce localized biofunctionalization, conductive patterns, sensing elements, microstructured interfaces, and topographical cues with high spatial precision. Such strategies are particularly promising for biosensors, lab-on-chip platforms, tissue-engineered scaffolds, drug-delivery systems, and flexible or implantable bioelectronic devices.

Future research should prioritize ink formulations combining aerosol stability, suitable rheology, biological functionality, and mild curing or cross-linking. Systematic process maps and standardized metrics are needed to compare resolution, throughput, material consumption, biological activity, reproducibility, scalability, and device performance. Machine-learning approaches may accelerate this progress by identifying suitable operating windows and supporting the simultaneous optimization of structural fidelity and biological response.

Overall, AJP is a promising but not yet fully mature biotechnology platform. Its development into a reliable fabrication tool will require advanced materials, application-specific process optimization, hybrid manufacturing strategies, and rigorous functional assessment in representative biological environments.

## Figures and Tables

**Table 1:** Overview of bioinks in aerosol jet printing.

Bioink/Material	Composition	Atomization	Resolution	Biological Outcome	Main Limitations
Collagen Type I	Pure collagen type I; also collagen-HAp composites	Mainly ultrasonic (UA); also pneumatic (PA)	~20–50 µm lines; multilayer dense scaffolds	Supports tissue engineering, cartilage/cornea-like constructs; enhanced piezoelectricity; good cell interaction	Collagen degradation during ultrasonic atomization; denaturation during prolonged printing; crosslinking complexity; scalability issues
Collagen Type II	Pure collagen type II	UA and PA	Dense multilayer structures; high-resolution layers	Mechanical properties comparable to cartilage; lower denaturation than type I	Process optimization still critical; scalability limitations
Recombinant Human Collagen III (RHCIII)	Recombinant collagen III	AJP (ShGF 60 cc/min, CGF 40 cc/min)	High-resolution transparent films	Corneal tissue engineering; 87.5% optical transmission; elasticity within corneal range	Partial denaturation during printing
Collagen + Silver Nanoparticles	Collagen/silver co-printed hybrid electrode	Co-printing AJP	Fine conductive electrode features	Flexible bioelectrodes; biocompatibility improved by increasing collagen fraction; conductivity ~10^−6^ Ω·m	Silver-related cytotoxicity; long-term biocompatibility concerns
Collagen + Hydroxyapatite (HAp)	Type I collagen + HAp	Ultrasonic	Hollow pillars; ~20–50 µm features	Bone tissue engineering; mimics bone composition	Ultrasonic degradation of collagen; CNC additives introduced cytotoxicity
Gelatin	Gelatin solution (collagen-derived)	Mainly ultrasonic	Lines down to 30 µm	Suitable for scaffolds, hydrogels, controlled-release systems	Strong swelling after deposition; coffee-ring effect; sensitive to process parameters
Silk Fibroin (SF)	Regenerated silk fibroin solution without additives	AJP; optimization of CGF/ShGF	~10 µm line width	High-resolution protein patterning; potential for tissue engineering and drug delivery	Difficult printability; narrow process window; challenging fabrication of thick 3D structures
BSA (Bovine Serum Albumin)	Fluorescently labeled BSA protein	UA and PA	10–20 µm lines and spots	Protein function retained after printing; microscale biofunctionalization	Overspray control required; formulation sensitive
DNA Bioinks	DNA ladders, biomolecular inks	UA and PA	10–20 µm features	DNA patterning for biosensors and biofunctional surfaces	High-molecular-weight DNA damaged by UA; preserved with PA
Horseradish Peroxidase (HRP)	Enzyme-based bioink	UA and PA	Microscale patterns	Retained enzymatic activity after printing and mild heating	Requires mild thermal treatment; formulation-dependent
Antibodies (anti-CEA), Streptavidin	Protein recognition molecules	Ultrasonic	Microscale immunoassay patterns	Maintained bioactivity; sub-ng/mL detection limits in biosensors	Mainly demonstrated for biosensing rather than tissue engineering
Alginate + Cells	Alginate hydrogel crosslinked by aerosolized CaCl_2_	Aerosol spraying crosslinking strategy	Thick porous scaffolds	~86% cell viability; sustained proliferation; interconnected pores	Mechanical properties remain lower than hard-tissue scaffolds

**Table 2:** Advantages and limitations of AJP in the biotechnology applications reviewed herein.

Application Area	Main Advantages of AJP	Main Limitations	Comparison with Other Techniques
Biosensors and Surface Biofunctionalization	High spatial resolution (10–20 μm); localized deposition of proteins, DNA and antibodies; compatibility with non-planar substrates; possibility to integrate biological and conductive materials within the same platform.	Potential biomolecule degradation during atomization; process optimization required to minimize overspray and preserve activity.	Superior resolution and conformal deposition compared with conventional IJP; less suited than lithography for large-scale manufacturing.
Microfluidics and Lab-on-Chip	Rapid prototyping; maskless fabrication; direct integration of microchannels, sensing elements and functional coatings; reduced dependence on cleanroom processing.	Resolution affected by overspray and droplet spreading; process repeatability strongly dependent on ink formulation and gas-flow tuning.	Faster and less expensive than conventional soft lithography; lower geometric accuracy than photolithographic approaches.
Cell Guidance and Tissue Patterning	Precise control of surface chemistry and topography; ability to create cell-adhesive and cell-repellent patterns; enhanced alignment and migration of cells.	Mostly limited to surface engineering rather than bulk tissue fabrication; long-term biological validation still limited.	Higher flexibility and design freedom than conventional coating approaches; complements rather than replaces scaffold-based bioprinting.
Neuroprosthetics and Bioelectronics	High-resolution deposition of conductive materials; fabrication of customized neural interfaces; integration with flexible substrates; rapid prototyping.	Requires optimization of conductive inks and post-processing; long-term implant stability remains under investigation.	Particularly advantageous over standard microfabrication for customization and prototyping; often combined with conventional microelectronics processes.
Surface Acoustic Wave (SAW) Devices	Direct fabrication of IDTs and sensing structures; reduced manufacturing complexity; compatibility with flexible and unconventional substrates.	Printed conductor performance may remain below lithographically fabricated devices; frequency limits associated with printing resolution.	Significant reduction of fabrication time and cost; lower precision than cleanroom lithography for high-frequency devices.
Drug Delivery Systems	Precise control of drug distribution; compatibility with thermally sensitive compounds; capability to fabricate customized dosage forms and microneedles.	Limited throughput; challenges in scaling production; need for careful control of morphology and solid-state properties.	More versatile than conventional pharmaceutical manufacturing for personalized formulations; lower throughput for industrial production.
Tissue Engineering Scaffolds	Ability to process biomaterials and composite formulations; controlled surface microstructuring; possibility to integrate biochemical and electrical functionalities.	Limited thickness per single pass; multiple deposition cycles often required; potential atomization-induced effects on proteins and cells.	Resolution typically higher than extrusion bioprinting; lower volumetric productivity for bulk scaffold fabrication.
3D Microstructured Architectures	Fabrication of pillars, lattices and high-aspect-ratio microstructures; excellent control of microscale geometries; suitable for multifunctional scaffolds.	Slow build-up of macroscopic volumes; strong dependence on viscosity, deposition regime and process tuning.	Outperforms IJP in feature definition and printable viscosity range; less efficient than extrusion-based systems for large-volume constructs.

## Data Availability

No new data were created or analyzed in this study. Data sharing is not applicable to this article.
